# Barcoded Pyrosequencing Reveals a Shift in the Bacterial Community in the Rhizosphere and Rhizoplane of *Rehmannia glutinosa* under Consecutive Monoculture

**DOI:** 10.3390/ijms19030850

**Published:** 2018-03-14

**Authors:** Linkun Wu, Jun Chen, Zhigang Xiao, Xiaocheng Zhu, Juanying Wang, Hongmiao Wu, Yanhong Wu, Zhongyi Zhang, Wenxiong Lin

**Affiliations:** 1 College of Life Sciences, Fujian Agriculture and Forestry University, Fuzhou 350002, China; wulinkun619@163.com (L.W.); chenjunfafu@163.com (J.C.); m18305987298@163.com (Z.X.); juanying020@163.com (J.W.); wuhongmiao2010@163.com (H.W.); wuyanhong_2016@163.com (Y.W.); 2 Fujian Provincial Key Laboratory of Agroecological Processing and Safety Monitoring, Fujian Agriculture and Forestry University, Fuzhou 350002, China; 3 Key Laboratory of Crop Ecology and Molecular Physiology (Fujian Agriculture and Forestry University), Fujian Province University, Fuzhou 350002, China; zyzhang@fafu.edu.cn; 4 Graham Centre for Agricultural Innovation (Charles Sturt University and NSW Department of Primary Industries), Charles Sturt University, Wagga NSW 2678, Australia; XZhu@csu.edu.au; 5 College of Crop Science, Fujian Agriculture and Forestry University, Fuzhou 350002, China

**Keywords:** *Rehmannia glutinosa*, consecutive monoculture, root-associated microbiome, deep pyrosequencing, beneficial rhizobacteria

## Abstract

The production and quality of *Rehmannia glutinosa* can be dramatically reduced by replant disease under consecutive monoculture. The root-associated microbiome, also known as the second genome of the plant, was investigated to understand its impact on plant health. Culture-dependent and culture-independent pyrosequencing analysis was applied to assess the shifts in soil bacterial communities in the rhizosphere and rhizoplane under consecutive monoculture. The results show that the root-associated microbiome (including rhizosphere and rhizoplane microbiomes) was significantly impacted by rhizocompartments and consecutive monoculture. Consecutive monoculture of *R. glutinosa* led to a significant decline in the relative abundance of the phyla *Firmicutes* and *Actinobacteria* in the rhizosphere and rhizoplane. Furthermore, the families *Flavobacteriaceae*, *Sphingomonadaceae*, and *Xanthomonadaceae* enriched while *Pseudomonadaceae*, *Bacillaceae*, and *Micrococcaceae* decreased under consecutive monoculture. At the genus level, *Pseudomonas*, *Bacillus*, and *Arthrobacter* were prevalent in the newly planted soil, which decreased in consecutive monocultured soils. Besides, culture-dependent analysis confirmed the widespread presence of *Pseudomonas* spp. and *Bacillus* spp. in newly planted soil and their strong antagonistic activities against fungal pathogens. In conclusion, *R. glutinosa* monoculture resulted in distinct root-associated microbiome variation with a reduction in the abundance of beneficial microbes, which might contribute to the declined soil suppressiveness to fungal pathogens in the monoculture regime.

## 1. Introduction

*Rehmannia glutinosa*, a perennial medicinal plant, belongs to the family *Scrophulariaceae* and has very widespread medicinal purposes. The tuberous root of *R. glutinosa* is one of the 50 fundamental herbs used in traditional Chinese medicine. It provides multiple pharmacological actions on the blood, immune, endocrine, cardiovascular, and nervous systems [1]. This species is produced mainly in Jiaozuo City, Henan Province, central China, known as the geo-authentic production zone with the most suitable soil and climate conditions. The quality of plants grown outside the geo-authentic areas cannot be assured. Meanwhile, consecutive monoculture of this plant in the same field can result in total crop failure of underground tubers. Compared with newly planted *R. glutinosa*, a two-year consecutive monoculture led to unexpanded tuberous roots with large numbers of adventitious fibrous roots (Figure S1) [2]. In addition, consecutive monoculture resulted in a significant increase in the abundance of soil-borne fungal pathogens, such as *Fusarium oxysporum* and *Aspergillus flavus* [2,3]. Therefore, commercial *R. glutinosa* are typically replanted every 15–20 years in the same field [4]. It is critical to understand the mechanisms that drive the replant disease of *R. glutinosa* to increase the efficiency and quality of production.

Numerous studies have indicated that replant disease is attributed to a deficiency in soil nutrients and the direct allelopathic autotoxicity of root exudates or plant residue [5,6]. However, our previous study has indicated that *R. glutinosa* consecutive monoculture did not lead to decreased soil organic matter and available nutrients [7]. Many studies have indicated that plants could produce toxic compounds that inhibit their own growth and reproduction [8,9]. For example, allelochemicals and/or autotoxic compounds such as β-cembrenediol, Di-n-hexyl Phthalate, and bis(2-propylheptyl) phthalate, which significantly reduce seedling growth, were found in flue-cured tobacco [9]. In contrast, indirect allelopathy is driven by variations in the soil microbial communities that are associated with root exudates [6,10,11]. Li et al. [10] reported indirect allelopathy of peanut (*Arachis hypogaea*) through the exudation of phenolic acids that induced the growth of pathogens and reduced the beneficial soil microorganisms. Zhou and Wu [12] indicated that *p*-coumaric acid influenced considerably the soil microbial communities and promoted the growth of pathogenic *Fusarium oxysporum* f.sp. *cucumerinum* Owen, which then resulted in replant disease of cucumber. Similar examples were also found in *Malus pumila*, *Radix pseudostellariae*, and *R. glutinosa* [2,13,14].

Previous studies using terminal restriction fragment length polymorphism (T-RFLP) and phospholipid fatty acid (PLFA) analyses demonstrated that consecutive monoculture of *R. glutinosa* led to significant changes in the soil microbial biomass and community structure [2,3]. However, the resolution and veracity of soil microbial identity are limited using T-RFLP and PLFA technologies [15]. Thus, a more reliable approach with higher resolution is required to improve the understanding of the dynamics of soil microbial communities. High-throughput pyrosequencing of 16S rRNA gene fragments has proven to be a powerful tool for in-depth sequencing and parallel analysis of soil and root microbial communities at a reduced sequencing error rate [16,17]. Illumina pyrosequencing was applied for the study of soil microbial communities under *R. glutinosa* monoculture [18]. However, the rhizoplane of plant root systems plays a selective gating role in microbiome recruitment from soil [19] depending on the root phenotypic traits [20]. This aspect was not studied in previous studies [18]. In addition, antagonistic activities of beneficial microbial biomarkers in disease-suppressive soils are still poorly understood [18]. Even though severe replant disease is observed in the field and confirmed by the detection of specific fungal pathogens (*F. oxysporum* and *A. flavus*) by quantitative PCR [3], little is known about the shifts in root-associated bacterial communities and their relationships with the increase of some specific soil-borne pathogens (i.e., *F. oxysporum* and *A. flavus*) under consecutive monoculture. Understanding the complex root-associated microbial communities from both rhizosphere and rhizoplane is important and useful for increasing soil health and improving plant performance.

In this study, barcoded pyrosequencing of 16S rRNA genes combined with a culture-dependent approach was applied to characterize the soil bacterial communities under *R. glutinosa* consecutive monoculture. We hypothesized that consecutive monoculture practice could alter the structure of bacterial communities in both the rhizosphere and rhizoplane with a reduction in the abundance of beneficial root-associated rhizobacteria showing antagonistic activities against *R. glutinosa* replant disease causal agents.

## 2. Results

### 2.1. Operational Taxonomic Unit (OTU) Cluster and Species Annotation

**Figure 1:**
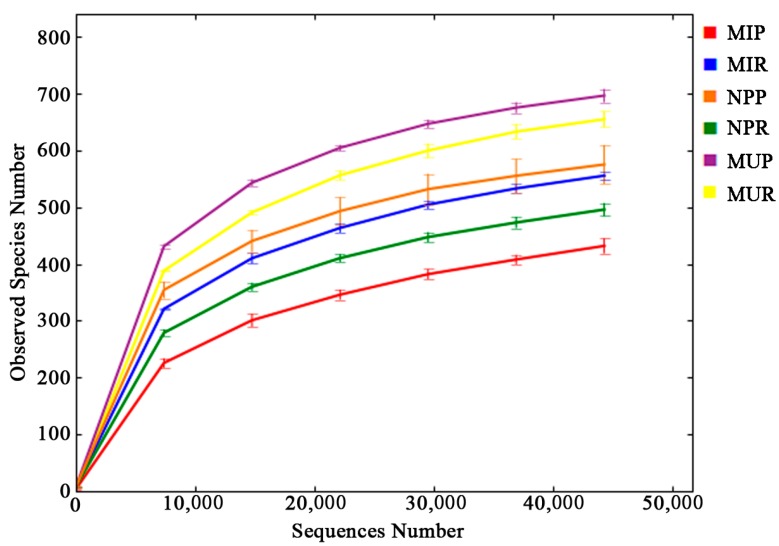
Rarefaction curves of bacterial communities based on observed operational taxonomic units (OTUs) at the 97% similarity cut-off level for individual samples. NPR, MUR, and MIR represent the rhizosphere soil from the newly planted and monocultured uninfected and infected plants, respectively. NPP, MUP, and MIP represent the rhizoplane soil from the newly planted and monocultured uninfected and infected plants, respectively.

Deep pyrosequencing of 16S rDNA amplicons was applied to assess the changes in the rhizosphere and rhizoplane bacterial communities under consecutive monoculture of *R. glutinosa*. A total of 995,947 effective tags with species annotation were obtained from 18 soil samples (as shown in Table S1) with an average of 55,330 effective tags per sample. Singletons accounted for 3.8% (37,683 tags) of the total tags and were removed from the dataset before further analyses (Figure S2). All libraries represented the bacterial communities well as the rarefaction curves were approaching plateaus (Figure 1). Based on 97% species similarity, 584, 740, 633, 649, 767, and 481 operational taxonomic units (OTUs) were obtained in the rhizosphere soil samples from the newly planted (NPR), monocultured uninfected (MUR), and monocultured infected (MIR) plants, and the rhizoplane soil samples from the newly planted (NPP), monocultured uninfected (MUP), and monocultured infected (MIP) plants, respectively (Figure S2). On average, 99.98%, 99.91%, 87.95%, 86.89%, 78.91%, and 18.75% of effective sequences from all 18 samples could be assigned to the phylum, class, order, family, genus, and species levels, respectively (Table S2). 

### 2.2. Alpha Diversity Analysis

The bacterial diversity and richness indices of different soil samples were calculated based on 44,256 sequences. In the rhizosphere, MUR showed the highest level of diversity (5.675) and highest species richness with observed species, Chao1, and ACE (abundance-based coverage estimator) indices at 654.7, 718.5, and 742.5 respectively. NPR had the lowest species richness and diversity compared with the other two rhizosphere soil groups (Table 1). Most of the richness and diversity indices showed significant differences among the three soil groups but no significant difference was observed in the ACE index between NPR and MIR (*p* > 0.05) (Table 1).

In the rhizoplane soil, there was no significant difference between MUP and NPP in terms of the Shannon diversity index and most of richness indices (Chao1 and ACE). Richness indices (including observed species and ACE) and Shannon diversity index were significantly higher in MUP than in MIP (*p* < 0.05) (Table 1). In addition, observed species and Shannon diversity index were significantly higher in the rhizoplane soil than in the rhizosphere soil of NP and MU plants (NPR vs. NPP and MUR vs. MUP). However, these two indices were higher in the rhizosphere soil of MI plants (Table S3).

### 2.3. Beta Diversity Analysis

**Figure 2:**
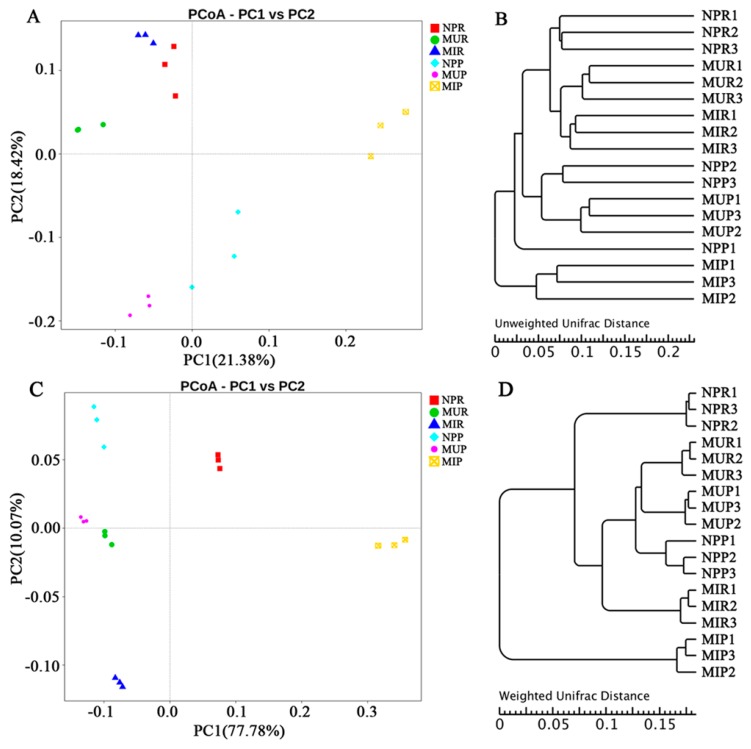
Principal coordinate analysis (PCoA) and unweighted pair-group method with arithmetic mean (UPGMA) dendrogram of bacterial communities based on unweighted (**A**,**B**) and weighted (**C**,**D**) Unifrac distance. NPR, MUR, and MIR represent the rhizosphere soil from the newly planted, monocultured uninfected and infected plants, respectively. NPP, MUP, and MIP represent the rhizoplane soil from the newly planted, monocultured uninfected and infected plants, respectively.

In this study, principal coordinate analysis (PCoA) and unweighted pair-group method with arithmetic mean (UPGMA) clustering based on unweighted UniFrac metric (UUF) and weighted UniFrac metric (WUF) were performed to investigate beta diversity patterns between complex bacterial communities (Figure 2). Both PCoA and UPGMA clustering based on UUF distances indicated that the microbiomes were separated by rhizocompartment (rhizosphere vs. rhizoplane) and longevity of monoculture (one-year vs. two-year monoculture) (Figure 2A,B). The first two axes PC1 and PC2 accounted for 21.38% and 18.42% of total variation, respectively. By contrast, PCoA and UPGMA clustering based on WUF distances showed a discernible separation among the newly planted (NP), two-year monocultured uninfected (MU), and monocultured infected (MI) plants (Figure 2C,D). The first two axes PC1 and PC2 accounted for 77.78% and 10.07% of total variation, respectively. Furthermore, non-metric multidimensional scaling (NMDS) ordinations (stress value = 0.101) showed that the plots monocultured for different years (one-year vs. two-year monoculture) separated across the first MDS axis and the rhizocompartments (except for MIP1) separated across the second MDS axis (Figure S3).

### 2.4. Venn Diagram and Similarity Analysis

Venn diagram analysis showed that the number of OTUs exclusively found in each group was much lower than that of shared OTUs in both rhizosphere and rhizoplane (Figure S4A,B). Meanwhile, the number of OTUs exclusively found in the rhizosphere or rhizoplane was much lower than that of OTUs shared between the rhizosphere and rhizoplane in the three different groups (Figure S4C–E). Permutational multivariate analysis of variance (permutational MANOVA) showed that the bacterial communities differed significantly (*p* < 0.05) between the rhizosphere and rhizoplane for all three different groups (NPR vs. NPP, MUR vs. MUP, MIR vs. MIP) (Table 2). In both the rhizosphere and rhizoplane, there was a significant difference between the newly planted and the monocultured infected groups (NPR vs. MIR, NPP vs. MIP). However, no significant difference was found between the newly planted and the monocultured uninfected groups (NPR vs. MUR, NPP vs. MUP) (Table 2). Furthermore, a two-way ANOSIM test of the pyrosequencing data consisting of the relative abundance of genera (logarithmic transformation and standardization, Bray–Curtis similarity) showed that the bacterial communities differed significantly among the three different groups (ANOSIM Global *R* = 1.0, *p* = 0.001) and between the rhizosphere and rhizoplane (ANOSIM Global *R* = 1.0, *p* = 0.003). Two-way ANOSIM analysis based on the presence/absence of genera (Bray-Curtis similarity) also showed the bacterial communities were significantly affected by the longevity of the monoculture (ANOSIM Global *R* = 0.735, *p* = 0.001) and rhizocompartment (ANOSIM Global *R* = 0.975, *p* = 0.001).

### 2.5. Shifts in Soil Bacterial Community Structure under Consecutive Monoculture

**Figure 3:**
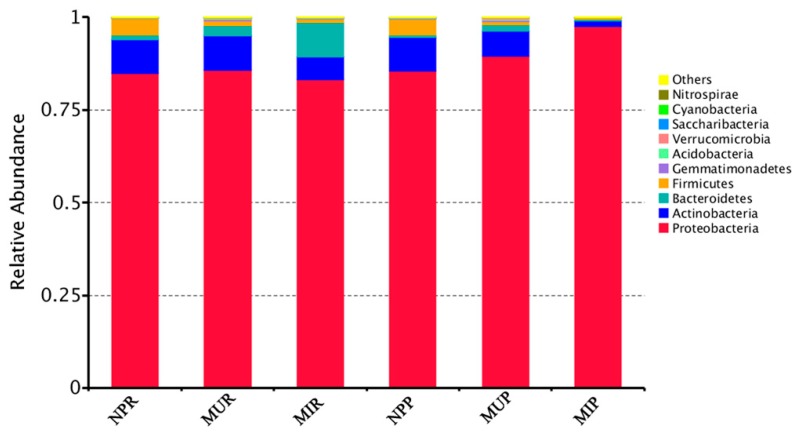
Relative abundances of the top 10 bacterial phyla in six different soil categories. NPR, MUR, and MIR represent the rhizosphere soil from the newly planted and monocultured uninfected and infected plants, respectively. NPP, MUP, and MIP represent the rhizoplane soil from the newly planted and monocultured uninfected and infected plants, respectively.

At the phylum level, the majority of the OTUs in all soil categories were assigned to *Proteobacteria* (83.2~97.6%), *Actinobacteria* (1.4~9.3%), *Bacteriodetes* (0.5~9.3%), and *Firmicutes* (0.3~4.5%) (Figure 3). However, clear trends in variation at the phylum level were observed between the NP group and the monocultured groups (NP vs. MU and NP vs. MI) (Figure 3).

*Firmicutes* was significantly (*p* < 0.05) decreased while *Bacteroidetes*, *Gemmatimonadetes*, *Acidobacteria*, and *Verrucomicrobia* were significantly (*p* < 0.05) increased in both rhizosphere and rhizoplane under consecutive monoculture (NPR vs. MUR, NPP vs. MUP, Figure 3). In addition, a higher abundance of *Proteobacteria* was detected in the monocultured rhizoplane (MUP) compared to NPP (Figure 3).

Both *Actinobacteria* and *Firmicutes* were significantly (*p* < 0.05) reduced in monocultured infected plants than in the newly planted plants (NPR vs. MIR, NPP vs. MIP) regardless of the rhizocompartment. More precisely, *Proteobacteria*, *Actinobacteria*, and *Firmicutes* were significantly (*p* < 0.05) reduced in the rhizosphere of the MI plant group (MIR) while *Bacteroidetes* and *Gemmatimonadetes* significantly (*p* < 0.05) increased in MIR compared to NPR (Figure 3). In the rhizoplane of the MI plant group (MIP), *Actinobacteria*, *Firmicutes*, *Gemmatimonadetes*, and *Acidobacteria* were significantly (*p* < 0.05) lower while *Proteobacteria* was significantly (*p* < 0.05) higher in comparison with NPP (Figure 3). Interestingly, the phylum *Firmicutes* was significantly (*p* < 0.05) reduced in both consecutive monoculture groups (MU and MI) regardless of the rhizocompartment (Figure 3).

**Figure 4:**
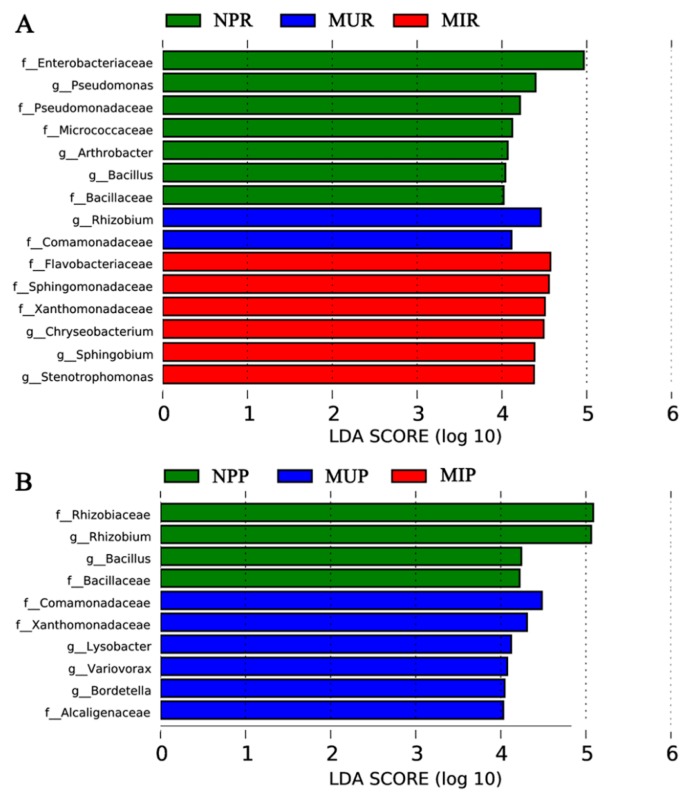
The linear discriminant analysis effect size (LEfSe) identified the most differentially abundant taxa between the newly planted and the two-year monocultured in the rhizosphere (**A**) and rhizoplane (**B**). Only taxa meeting a linear discriminant analysis (LDA) significant threshold >4 are shown. The prefixes ‘f_’ and ‘g_’ mean family level and genus level, respectively. NPR, MUR, and MIR represent the rhizosphere soil from the newly planted and monocultured uninfected and infected plants, respectively. NPP, MUP, and MIP represent the rhizoplane soil from the newly planted and monocultured uninfected and infected plants, respectively.

Furthermore, the linear discriminant analysis effect size (LEfSe) method was applied to analyze the differential abundance of soil bacterial taxa at the family and genus levels between the newly planted and consecutively monocultured groups, as shown by the linear discriminant analysis (LDA) score in Figure 4. The results indicated that microbial dysbiosis was prevalent in *R. glutinosa* rhizosphere (Figure 4A) and rhizoplane (Figure 4B) under consecutive monoculture. In the NP rhizosphere, the dominant microbial taxa included the families of *Enterobacteriaceae*, *Pseudomonadaceae*, *Micrococcaceae*, and *Bacillaceae* and the genera *Pseudomonas*, *Bacillus*, and *Arthrobacter*. In contrast, the families *Comamonadaceae* (in MUR) and *Flavobacteriaceae*, *Sphingomonadaceae*, and *Xanthomonadaceae* (in MIR) were enriched in the rhizosphere of the two-year monocultured plants. Genera *Rhizobium* (in MUR) and *Chryseobacterium*, *Sphingobium*, and *Stenotrophomonas* (in MIR) were enriched in the rhizosphere of the two-year monocultured plants (Figure 4A). In the rhizoplane, the families *Rhizobiaceae* and *Bacillaceae* and genera *Rhizobium* and *Bacillus* were significantly more abundant in the newly planted while the families *Comamonadaceae*, *Xanthomonadaceae*, and *Alcaligenaceae* and genera *Lysobacter*, *Variovorax*, and *Bordetella* were significantly more abundant in the two-year consecutive monoculture (Figure 4B).

### 2.6. Isolation of Pseudomonas and Bacillus and Their Antagonistic Activities Assessment

**Figure 5:**
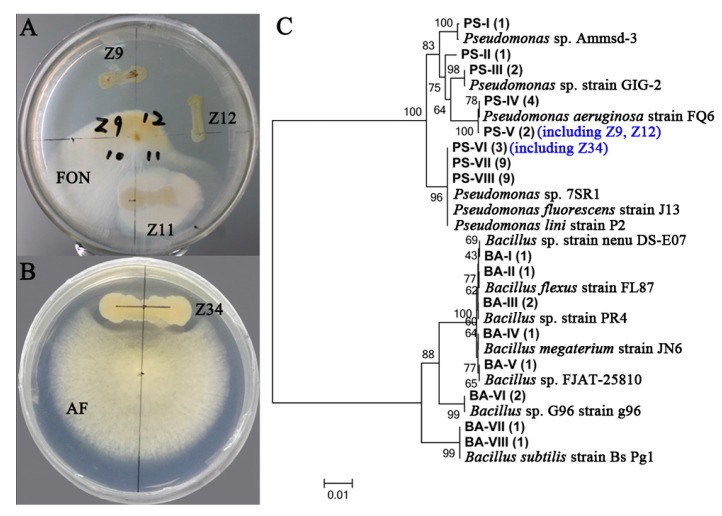
Assessment of antagonistic activity (**A**,**B**) and hierarchical clustering of 16S rDNA genes (**C**) of *Pseudomonas* spp. and *Bacillus* spp. isolated from the newly planted soil. (**A**) Strain Z9 (GenBank: MH037322) and Z12 (GenBank: MH037323) but not Z11 have antagonistic activity against *F. oxysporum* (FON). (**B**) Strain Z34 (GenBank: MH037324) has antagonistic activity against *A. flavus* (AF). (**C**) Different *Pseudomonas* and *Bacillus* species were used as references. Eight clusters were identified for both *Pseudomonas* (PS-I to PS-VIII) and *Bacillus* (BA-I to BA-VIII). Numbers of isolates of each cluster are indicated in parentheses. Three isolates (Z9, Z12 and Z34) with antagonistic activities in A and B are presented in the phylogenetic tree with blue font.

Both *Pseudomonas* and *Bacillus*, commonly used as biocontrol agents of plant pathogens, were enriched in the newly planted soil through LEfSe analysis. Therefore, *Pseudomonas* spp. and *Bacillus* spp. were isolated from the newly planted soil using two selective screening agar to evaluate their roles in maintaining soil health. *Pseudomonas* spp. and *Bacillus* spp. were co-cultured with two main causal agents of *R. glutinosa* root rot disease including *Fusarium oxysporum* and *Aspergillus flavus* [3]. Strong antagonistic activities were detected in many *Pseudomonas* spp. and *Bacillus* spp. (Figure 5A,B), which suggest these two genera are critical for maintaining soil health. The identities of the antagonistic isolates were further confirmed with Sanger sequencing on the 16S rRNA gene region (Figure 5C).

## 3. Discussion

Replant disease is a very common but complicated phenomenon in the agricultural production of many crops, especially for Chinese medicinal plants. Our field experiment showed that consecutive monoculture practice has a profound effect on the growth and health of *R. glutinosa*, which results in poor plant performance and serious root rot disease (Figure S1). Recently, numerous studies have indicated that indirect allelopathy through modifications in soil microbial community is closely related to replant disease in agriculture and horticulture [10,21]. The complex plant-associated microbial community is crucial for soil health and plant growth, which is also referred to as a second genome to a plant [22,23]. The rhizosphere is the battlefield for soil-borne pathogens and beneficial microorganisms [24], while the rhizoplane, or root tissue surface, plays a selective gating role in root microbiome assembly from the soil [19]. In this study, parallel pyrosequencing was applied to explore the shifts in bacterial communities in the rhizosphere and rhizoplane of *R. glutinosa* and highlighted that consecutive monoculture of *R. glutinosa* significantly restructures the bacterial communities in both the rhizosphere and the rhizoplane. PCoA analysis based on weighted and unweighted UniFrac distances highlighted distinct differences in bacterial community structure among the soils sampled from the newly planted, the monocultured uninfected and infected plants (Figure 2). Moreover, PCoA also suggested significant differences in soil bacterial communities between rhizocompartments (Figure 2), which is well aligned with previous studies on *Oryza sativa* and *Solanum lycopersicum* [19,25].

A high level of microbial diversity is generally considered as an important parameter for soil health [26]. For example, a reduction in species diversity with the extended monoculture period was reported in *Solanum tuberosum* [27]. However, this study suggested most of richness and diversity indices were significantly higher in the two-year monocultured soil than in the newly planted soil in the rhizosphere (NPR vs. MUR; NPR vs. MIR) and was similar in the rhizoplane (NPP vs. MUP) (Table 1). This result is aligned with previous research on *Vanilla planifolia* [28]. Six-year monoculture resulted in a significantly higher species diversity in the rhizosphere [28]. In addition, Xiong et al. [28] also suggested that a low bacterial diversity and high fungal diversity could associate with the suppression of vanilla *Fusarium* wilt disease in disease-suppressive soil. Therefore, the discrepancy between these different studies might be due not only to the longevity of monoculture but also to the different soil environmental conditions, host types, rhizocompartment, etc. More recent research suggested that the key component of soil ecosystem functions is not taxonomic diversity but rather functional diversity [29,30].

Taxonomic analysis showed that in both the rhizosphere and rhizoplane, the relative abundance of *Actinobacteria* was significantly lower in MI and *Firmicutes* was significantly lower in MU and MI when compared with NP (Figure 3). This is in line with previous findings where members of *Firmicutes* and *Actinobacteria* were consistently associated with pathogen antagonism and soil disease suppression [31,32,33]. Xiong et al. [28] compared microbial communities in suppressive- and conducive-soils associated with *Fusarium* wilt disease and found that *Actinobacteria* and *Firmicutes* were strongly enriched in the suppressive soil in a vanilla long-term continuous cropping system. Yang et al. [18] indicated that some probiotic bacteria belonging to *Actinobacteria* significantly decreased with the extended monoculture of *R. glutinosa*. In addition, disease suppression that is induced by bioorganic fertilizer application in *Nicotiana tabacum* and *Persea americana* was associated with an increase in multiple beneficial strains such as *Actinobacteria*, *Firmicutes*, etc. [34,35]. A previous study on banana Panama disease caused by *Fusarium oxysporum* f. sp. *cubense* showed that *Firmicutes* and *Actinobacteria* were more abundant in disease-suppressive soil while *Verrucomicrobia*, *Bacteroidetes*, *Proteobacteria*, and *Gemmatimonadetes* were more abundant in diseased soil [32]. In this study, we also found *Bacteroidetes*, *Gemmatimonadetes*, *Verrucomicrobia*, etc. to be significantly increased while *Firmicutes* and *Actinobacteria* were decreased under consecutive monoculture (Figure 3).

The LEfSe analysis indicated a concomitant increase in the families of *Flavobacteriaceae* and *Sphingomonadaceae* with the consecutive monoculture of *R. glutinosa* (Figure 4). Similarly, *Flavobacteriaceae* and *Sphingomonadaceae* were significantly more abundant in 4- and 15-year monocropped cotton rhizosphere soil compared to fallow soil [36]. Members of *Flavobacteriaceae* and *Sphingomonadaceae* frequently synthesize cellulose-, pectin-, xylan-, and chitin-degrading enzymes to decompose dead plants resulting from fungal invasion [36,37,38]. Therefore, the concomitant increase in the members of *Flavobacteriaceae* and *Sphingomonadaceae* in the soil could be important indicators of root damage by soil fungal pathogens [36,38]. Wu et al. [3] reported the high abundance of soil fungal pathogens *Fusarium oxysporum* and *Aspergillus flavus* under consecutive monoculture of *R. glutinosa*. Thus, the increase in *Flavobacteriaceae* and *Sphingomonadaceae* detected in this study might be caused by the *Fusarium oxysporum* and *Aspergillus flavus* invasion in *R. glutinosa*. Furthermore, it was found that the genus *Stenotrophomonas* was enriched in the consecutively monocultured soil, especially in the rhizosphere of infected plants (MIR) (Figure 4). Tan et al. [39] also found relatively higher proportions of genera such as *Stenotrophomonas*, *Sphingobium*, and *Erwinia*, etc. in the rhizosphere of root-rot *Panax notoginseng* under consecutive monoculture. In contrast, it was reported that several members of *Stenotrophomonas* could reduce the ecotoxicity of contaminants in the soil, promote plant growth, or be biological control agents of plant pathogens [40,41,42]. Therefore, further work is needed to study the roles of *Stenotrophomonas* in the rhizosphere in *R. glutinosa* monoculture regimes.

Besides, LEfSe analysis indicated that *Enterobacteriaceae*, *Pseudomonadaceae*, *Bacillaceae*, *Micrococcaceae*, *Pseudomonas*, *Bacillus*, and *Arthrobacter* were enriched in the newly planted group (Figure 4), possibly serving as important indicators for soil disease suppression. Many members of the *Enterobacteriaceae*, *Pseudomonadaceae* and *Bacillaceae* families have been reported as biocontrol agents capable of suppressing fungal and insect growth [43,44,45,46]. Mendes et al. [31] deciphered the rhizosphere microbiome for disease-suppressive bacteria and indicated that the relative abundances of *Pseudomonadaceae* and *Pseudomonas* species were positively correlated with disease suppression of sugar beet. In addition, the abundance of the genera *Pseudomonas*, Gp5, etc. were positively (*p* < 0.05) correlated with *Fusarium*-wilt suppression of banana [47]. Similarly, beneficial bacterial populations *Pseudomonas* and *Bacillus* in the rhizosphere were significantly reduced after apple replanting [48]. Furthermore, Xue et al. [32] highlighted that *Bacillus* was the top contributor to the community differences between disease-suppressive soil and diseased soil, and the family of *Bacillaceae* was reported as the dominant component of the ‘core microbiome’ from the disease-suppressive soil. Xiong et al. [49] found that the abundance of beneficial bacteria (i.e., *Bacillus*, etc.) significantly decreased along the years of *V. planifolia* monoculture, which might lead to the soil weakness and vanilla stem wilt disease under long-term monoculture. Our study (Figure 4) also coincides with previous studies, which suggested the important roles of the members of *Micrococcaceae*, such as *Arthrobacter* species, in soil disease suppression [34,43,50,51]. Despite these interpretations, there is a great need to explore the interactions between soil pathogenic and beneficial microbes *in situ* under *R. glutinosa* monoculture because microbial interactions in the phytosphere are crucial for plant health [46].

Members of *Pseudomonas* and *Bacillus* have been extensively used as potential antagonistic agents against various plant pathogens [52]. Grosch et al. [53] found that *Pseudomonas fluorescens* B1 could be selected as an effective biological control agent against *Rhizoctonia solani* in lettuce and potato plants. Zhang et al. [54] demonstrated that *Bacillus subtilis* N11-fortified bioorganic fertilizer can effectively control banana wilt. This study highlighted that many *Pseudomonas* spp. and *Bacillus* spp. isolates from the newly planted soil showed strong antagonistic activities against two main fungal pathogens (i.e., *F. oxysporum* and *A. flavus*) of *R. glutinosa* root rot disease (Figure 5). Moreover, a biocontrol test in a previous study showed that antagonistic *Pseudomonas* spp. isolated from the rhizosphere of *R. glutinosa* could effectively protect *R. glutinosa* plants against *F. oxysporum* infection [2]. Our previous study also showed that the abundance of antagonistic *Pseudomonas* spp. against *F. oxysporum* gradually decreased [2], while the fungal pathogens such as *F. oxysporum* and *A. flavus* increased under *R. glutinosa* consecutive monoculture [2,3]. Therefore, the decrease in beneficial bacteria (i.e., *Pseudomonas* and *Bacillus*) with the antagonistic activities against pathogens might contribute to the declined soil suppressiveness to fungal invasion in consecutive monoculture regimes. The isolation and biocontrol potential assessment of members of *Commamonadaceae* and *Rhizobiaceae* are now underway.

## 4. Materials and Methods

### 4.1. Field Experiment and Soil Sampling

The *R. glutinosa* cultivar “85-5” is widely planted on a large-scale in the geo-authentic production zones and was selected as the experimental material. The experiment was conducted at Jiaozuo City, Henan Province, China (34°56′ N, 112°58’ E), which has an annual mean temperature of 14.3 °C and an annual mean precipitation of 552 mm. The experiment was conducted in a single field site that had not been planted with *R. glutinosa* for more than 10 years to exclude the effects of preceding residues of this plant and to ensure the uniform growth condition among treatments. The physicochemical properties of the soil were as follows: sandy loam soil, soil organic matter 12.52 g·kg^−1^, pH 7.43, total nitrogen 0.51 g·kg^−1^, available nitrogen 23.41 mg·kg^−1^, total phosphorus 1.46 g·kg^−1^, available phosphorus 51.36 mg·kg^−1^, total potassium 6.98 g·kg^−1^, and available potassium 223.89 mg·kg^−1^. Two cropping patterns of *R. glutinosa* were performed including the newly planted and the two-year monocultured. The two-year monocultured *R. glutinosa* was planted on April 15 of 2015 and harvested on October 30 of 2015. The paddock was then kept fallow until replanting on April 15 of 2016 and soil samples collected in the second year were kept at −80 °C for further analysis. For the newly planted group, *R. glutinosa* was planted on April 15 of 2016. They were subjected to the same fertilization and field management during the entire experimental period.

Three groups of plants were separated for analysis according to their growth status including one newly planted groups and two monocultured groups. The newly planted group (NP) was the plants that were grown in the paddocks that had been free of *R. glutinosa* for at least 10 years. These plants are featured with normally expanded tuberous roots. The monocultured uninfected group (MU) is defined as the two-year monocultured plants without root rot disease symptoms and containing large numbers of adventitious fibrous roots, while the monocultured infected group (MI) refers to two-year monocultured plants with root rot disease symptoms (Figure S1).

Soils from two root-associated compartments—rhizosphere (R) and rhizoplane (P)—were collected (as shown in Figure S5) for each of the three groups on 15 July 2016, using a modification of the method previously described by Edwards et al. [19]. In brief, *R. glutinosa* plants were carefully uprooted and shaken to remove loosely attached soil on the tuberous roots. The tuberous roots with tightly attached soil were added into a 50 mL centrifuge tube with 20 mL sterile water. After vortex mixing at 200 rpm for 20 min, the washed roots were removed and the soil suspension was centrifuged at 11,000 rpm for 10 min. The deposit was kept as the rhizosphere compartment. The clean roots were washed twice more using sterile water and placed into a new 50 mL centrifuge tube with 20 mL sterile water. After 5 min of sonication, the suspension was centrifuged at 11,000 rpm for 10 min. The deposit was kept as the rhizoplane compartment. In total, six soil categories were analyzed with three replications: NP rhizosphere soil (NPR), MU rhizosphere soil (MUR), MI rhizosphere soil (MIR), NP rhizoplane soil (NPP), MU rhizoplane soil (MUP), and MI rhizoplane soil (MIP).

### 4.2. Soil DNA Extraction and PCR Amplification

Total DNA was extracted from soil samples using a BioFast soil Genomic DNA Extraction kit (BioFlux, Hangzhou, China) following the manufacturer’s instructions and further characterized by electrophoresis. DNA concentration was determined by Nanodrop 2000C Spectrophotometer (Thermo Scientific, Waltham, MA, USA) and adjusted to 1 ng/µL. Variable regions 5 to 7 (V5–V7) of bacterial 16S rRNA gene were selected and amplified with the specific primers 799F (5’-AACMGGATTAGATACCCKG-3’) and 1193R (5’-ACGTCATCCCCACCTTCC-3’) with the barcode [55]. This primer pair is featured with low co-amplification levels of plastid and mitochondrial sequences and high retrieval of bacterial reads, which can significantly reduce noise from trace amounts of decaying root, eukaryotic organisms, and root border cells [55]. All polymerase chain reaction (PCR) reactions were carried out using Phusion^®^ High-Fidelity PCR Master Mix with GC Buffer (New England Biolabs, Ipswich, MA, USA) and high-fidelity polymerase (New England Biolabs).

### 4.3. Library Preparation and Illumina Sequencing

PCR products with the unique barcode from each soil sample were equally mixed and then purified with Qiagen Gel Extraction Kit (Qiagen, Hilden, Germany) for parallel pyrosequencing. Library construction was performed with a TruSeq® DNA PCR-Free Sample Preparation Kit (Illumina, San Diego, CA, USA). The library quality was assessed on the Qubit@ 2.0 Fluorometer (Thermo Scientific) and Agilent Bioanalyzer 2100 system (Agilent Technologies, Santa Clara, CA, USA) Pyrosequencing was carried out on an Illumina HiSeq2500 platform and 250 bp paired-end reads were generated.

### 4.4. Operational Taxonomic Unit (OTU) Cluster and Species Annotation

Paired-end reads were assigned to each sample based on the unique barcode and then merged to obtain raw tags using FLASH (V1.2.7, Fast Length Adjustment of SHort reads [56]). After data filtration and chimera removal, effective tags (SRA accession: SRP134246) were obtained for further analysis. The effective tags were binned into OTUs using a 97% identity threshold. Taxonomy was assigned to bacterial OTUs by using the RDP classifier for each representative sequence against the GreenGene database [57,58].

### 4.5. Alpha Diversity and Beta Diversity Analyses

Alpha diversity and beta diversity analyses were carried out based on the normalized data. Alpha diversity indices were calculated based on 44,256 sequences with QIIME (Version 1.7.0, Quantitative Insights Into Microbial Ecology) [59] to analyze the richness and species diversity for each sample, including Observed species, Chao1, ACE, Shannon indices. Principal coordinate analysis (PCoA) and unweighted pair-group method with arithmetic mean (UPGMA) clustering based on weighted and unweighted UniFrac distances were performed to investigate beta-diversity patterns between complex bacterial communities. The UniFrac distance measures the phylogenetic relatedness of whole communities. The weighted UniFrac (WUF, quantitative measure) metric takes the abundance of taxa into consideration whereas the unweighted UniFrac (UUF, qualitative measure) assesses the presence/absence status of taxa and is thus more sensitive to rare taxa. Furthermore, non-metric multidimensional scaling (NMDS) ordinations were used to evaluate relative similarities of microbial composition. NMDS is a more robust method for investigating the microbial community variation when examining non-linear data, which use an iterative algorithm to successively refine sample positions in the ordination with Kruskal’s stress value used as a measure of goodness of fit [60]. A stress value less than 0.1 indicates an accurate and reliable ordination with a low probability of misinterpretation.

### 4.6. Statistical Analyses of Pyrosequencing Data

One-way analysis of variance (ANOVA) followed by the Tukey’s test (*p* < 0.05, *n* = 3) was carried out for multiple comparisons of the relative abundance of the bacterial groups and alpha diversity indices. Two-way crossed analysis of similarity (two-way ANOSIM) was used to investigate the effects of longevity of monoculture (NP vs. MU vs. MI) and rhizocompartment (R vs. P) on soil bacterial communities of *R. glutinosa*. The linear discriminant analysis (LDA) effect size (LEfSe) method was applied to examine significantly differentially bacterial taxa between three different groups with a threshold logarithmic LDA score higher than 4.0 [61].

### 4.7. Isolation of Pseudomonas spp. and Bacillus spp. and Their Antagonistic Activities Assessment

*Pseudomonas* spp. and *Bacillus* spp. were isolated from the newly planted soil using *Pseudomonas* and *Bacillus* selective isolation agar, respectively [62,63]. Isolated colonies from these two genera were co-cultured with two main soil-borne pathogens *Fusarium oxysporum* and *Aspergillus flavus* to evaluate their antagonistic activities against these two fungal causal agents of *R. glutinosa* root rot diseases [3]. In detail, single colonies from the selective isolation agar were inoculated at the periphery of potato-dextrose agar (PDA) plates and incubated for 2 days at 28 °C and 37 °C for *Pseudomonas* spp. and *Bacillus* spp., respectively. Then, *F. oxysporum* or *A. flavus* was transferred to the center of the PDA plates and incubated for an additional 4 days to observe antagonistic activities. The DNA of antagonistic bacteria was extracted and used for amplification with primers 357F (5’-CTCCTAGGGAGGCAGCAG-3’) and 1492r (5’-GGTTACCTTGTTACGACTT-3’) for Sanger sequencing.

## 5. Conclusions

**Figure 6:**
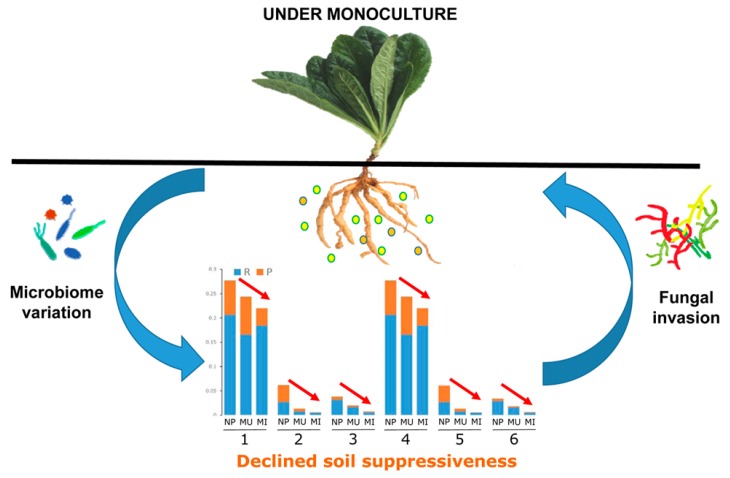
Schematic representation of the proposed interactions between root-associated microbes and *R. glutinosa* under consecutive monoculture. NP, MU, and MI represent the newly planted, monocultured uninfected and infected plants, respectively. R and P represent rhizosphere and rhizoplane soils, respectively. 1~6 represent the families of *Pseudomonadaceae*, *Bacillaceae*, and *Micrococcaceae*, and the genera of *Pseudomonas*, *Bacillus*, and *Arthrobacter*, respectively. Color circles represent root exudates released by *R. glutinosa*. Red arrows indicate the sum of above-mentioned taxa (1~6) gradually decreased with extended monoculture.

Our results demonstrated that *R. glutinosa* consecutive monoculture clearly affected the root-associated bacterial communities in both the rhizosphere and rhizoplane. It was found that the predominant taxa in newly planted soil significantly decreased under consecutive monoculture, including the phyla *Firmicutes* and *Actinobacteria* and the families *Pseudomonadaceae*, *Bacillaceae*, and *Micrococcaceae*, etc. In particular, the sum of the rhizosphere and rhizoplane abundance of several members (i.e., *Pseudomonas*, *Bacillus*, *Arthrobacter*) with antagonistic activities against pathogens gradually decreased with extended monoculture, which might lead to the loss of soil disease suppressiveness (Figure 6). Further studies on the manipulation of the root-associated microbiome and recruitment of beneficial microorganisms (i.e., *Pseudomonas* spp. and *Bacillus* spp.) under field conditions may give a promising avenue for the biocontrol of replant disease of *R. glutinosa*.

## Figures and Tables

**Table 1:** Species richness and diversity indices in different soil samples.

Categories	Observed Species	Chao1	ACE	Shannon
**Rhizosphere**				
NPR	495.7 ^c^	566.4 ^c^	593.7 ^b^	5.042 ^c^
MUR	654.7 ^a^	718.5 ^a^	742.5 ^a^	5.675 ^a^
MIR	555.3 ^b^	628.3 ^b^	651.9 ^b^	5.493 ^b^
**Rhizoplane**				
NPP	575.0 ^b^	634.3 ^a^	652.6 ^ab^	5.735 ^a^
MUP	696.3 ^a^	755.9 ^a^	766.7 ^a^	5.975 ^a^
MIP	431.7 ^c^	615.6 ^a^	565.8 ^b^	2.496 ^b^

NPR, MUR, and MIR represent the rhizosphere soil from the newly planted, monocultured uninfected and infected plants, respectively. NPP, MUP, and MIP represent the rhizoplane soil from the newly planted, monocultured uninfected and infected plants, respectively. Different letters in the columns show significant differences between the different treatments in the rhizosphere (NPR vs. MUR vs. MIR) or rhizoplane (NPP vs. MUP vs. MIP), determined by Tukey’s test (*p* < 0.05).

**Table 2:** Permutational multivariate analysis of variance (permutational MANOVA) to investigate the differences in bacterial communities between different soil categories.

Pairwise Comparison	*R*^2^	*p*-Value *
**Rhizosphere**		
NPR-MUR	0.9778	0.1000
NPR-MIR	0.9735	0.0014
MUR-MIR	0.9340	0.0014
**Rhizoplane**		
NPP-MUP	0.9158	0.1000
NPP-MIP	0.9867	0.0014
MUP-MIP	0.9929	0.0083
**Rhizosphere vs. rhizoplane**		
NPR-NPP	0.9580	0.0014
MUR-MUP	0.9276	0.0014
MIR-MIP	0.9913	0.0417

*R*^2^ represents the ratio of between-group variance to total variance. * Significant differences are indicated by bold *p*-values.

## Supplementary Materials

The following are available online at http://www.mdpi.com/1422-0067/19/3/850/s1.

## Abbreviations

T-RFLP	terminal restriction fragment length polymorphism
PLFA	phospholipid fatty acid
OTU	operational taxonomic unit
NPR	rhizosphere soil from the newly planted plants
MUR	rhizosphere soil from monocultured uninfected plants
MIR	rhizosphere soil from monocultured infected plants
NPP	rhizoplane soil from the newly planted plants
MUP	rhizoplane soil from monocultured uninfected plants
MIP	rhizoplane soil from monocultured infected plants
PCoA	principal coordinate analysis
UPGMA	unweighted pair-group method with arithmetic means
WUF	weighted UniFrac metric
UUF	unweighted UniFrac metric
NMDS	non-metric multidimensional scaling
ANOVA	one-way analysis of variance
ANOSIM	analysis of similarity
LDA	linear discriminant analysis
LEfSe	linear discriminant analysis effect size
PDA	potato-dextrose agar
