# Supplementary material for: Barcoded Pyrosequencing Reveals a Shift in the Bacterial Community in the Rhizosphere and Rhizoplane of Rehmannia glutinosa under Consecutive Monoculture
Source: Int J Mol Sci. 2018 Mar 14;19(3):850. doi: 10.3390/ijms19030850 (PMC5877711; doi:10.3390/ijms19030850)
Supplement: Supplementary file 1 [file ijms-19-00850-s001.pdf]

International Journal of
Molecular Sciences

MDPI

Barcoded Pyrosequencing Reveals a Shift in the Bacterial Community in the Rhizosphere and Rhizoplane of *Rehmannia glutinosa* under Consecutive Monoculture

Linkun Wu [1,2], Jun Chen [1,3], Zhigang Xiao [1,2], Xiaocheng Zhu [4,5], Juanying Wang [1,2], Hongmiao Wu [1,2], Yanhong Wu [1,2], Zhongyi Zhang [3,5] and Wenxiong Lin [2,3,*]

[1] College of Life Sciences, Fujian Agriculture and Forestry University, Fuzhou 350002, China; wulinkun619@163.com (L.W.); chenjunfafu@163.com (J.C.); m18305987298@163.com (Z.X.); juanying020@163.com (J.W.); wuhongmiao2010@163.com (H.W.); wuyanhong_2016@163.com (Y.W.)
[2] Fujian Provincial Key Laboratory of Agroecological Processing and Safety Monitoring, Fujian Agriculture and Forestry University, Fuzhou 350002, China
[3] Key Laboratory of Crop Ecology and Molecular Physiology (Fujian Agriculture and Forestry University), Fujian Province University, Fuzhou 350002, China; zyzhang@fafu.edu.cn
[4] Graham Centre for Agricultural Innovation (Charles Sturt University and NSW Department of Primary Industries), Charles Sturt University, Wagga NSW 2678, Australia; XZhu@csu.edu.au
[5] College of Crop Science, Fujian Agriculture and Forestry University, Fuzhou 350002, China
* Correspondence: lwx@fafu.edu.cn; Tel.: +86-591-8376-9440

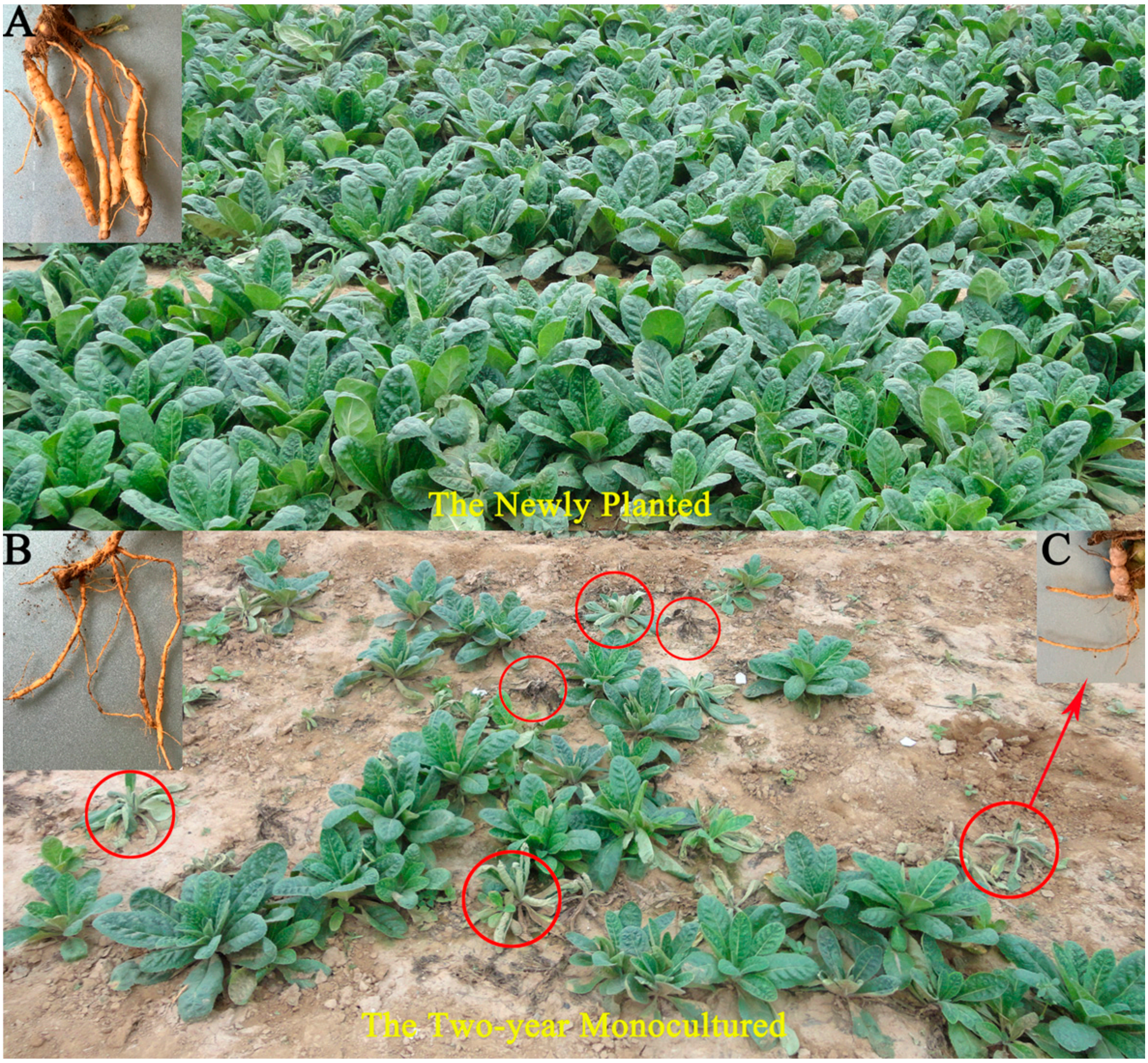

Figure S1. Photographs of above and below ground components of *Rehmannia glutinosa*. A, B and C represents the close-up views of the tuber roots of the newly planted plants (NP), the two-year monocultured uninfected plants (MU) and the two-year monocultured infected plants (MI). The two-year monocultured uninfected plants (MU) displayed large numbers of adventitious fibrous roots though without obvious symptoms of infection (B). The two-year monocultured infected plants (MI) displayed earlier wilt of the above ground plant parts (red circles), showing symptoms of infection in both leaf and tuberous roots (C).

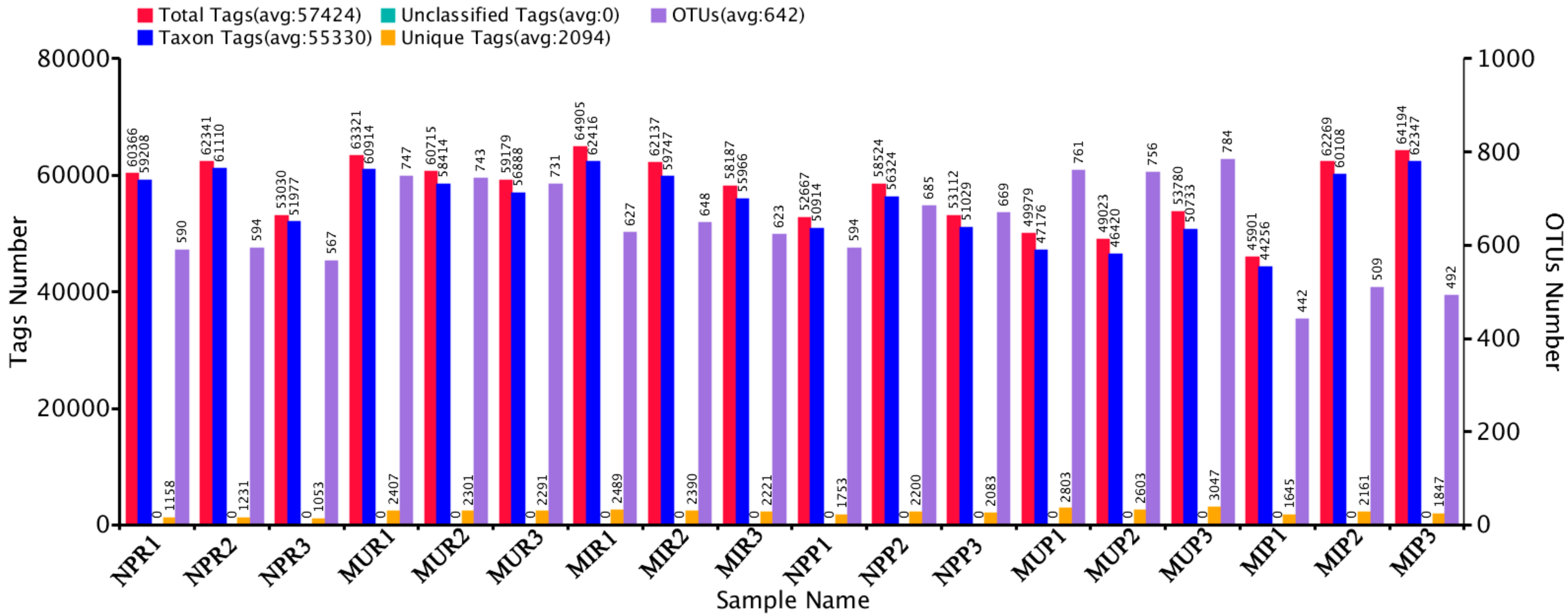

Figure S2. Statistics of OTU cluster and species annotation for individual samples. Total Tags (red columns): represent the total numbers of effective tags; Unique Tags (orange columns): represent the total numbers of singletons which were removed from the dataset before further analysis; Taxon Tags (blue columns): represent the total numbers of tags subjected to OTU cluster and with species annotation; Unclassified Tags (green columns): represent the total numbers of tags without species annotation; OTUs (purple columns): represent the OTUs numbers for each sample. NPR, MUR and MIR represent the rhizosphere soil from the newly planted, monocultured uninfected and infected plants, respectively. NPP, MUP and MIP represent the rhizoplane soil from the newly planted, monocultured uninfected and infected plants, respectively.

International Journal of
Molecular Sciences

MDPI

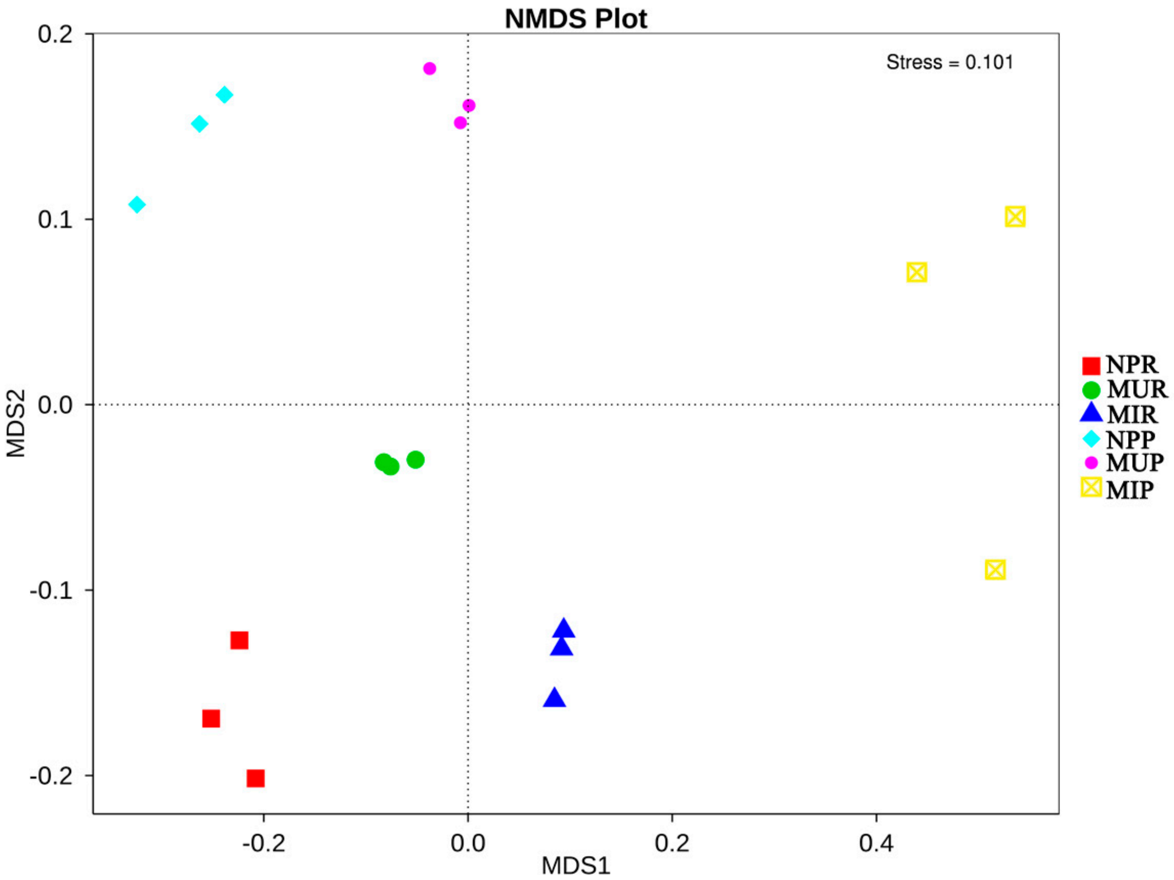

Figure S3. Non-metric multidimensional scaling (NMDS) ordinations analysis of bacterial communities among different soil categories. NPR, MUR and MIR represent the rhizosphere soil from the newly planted, monocultured uninfected and infected plants, respectively. NPP, MUP and MIP represent the rhizoplane soil from the newly planted, monocultured uninfected and infected plants, respectively.

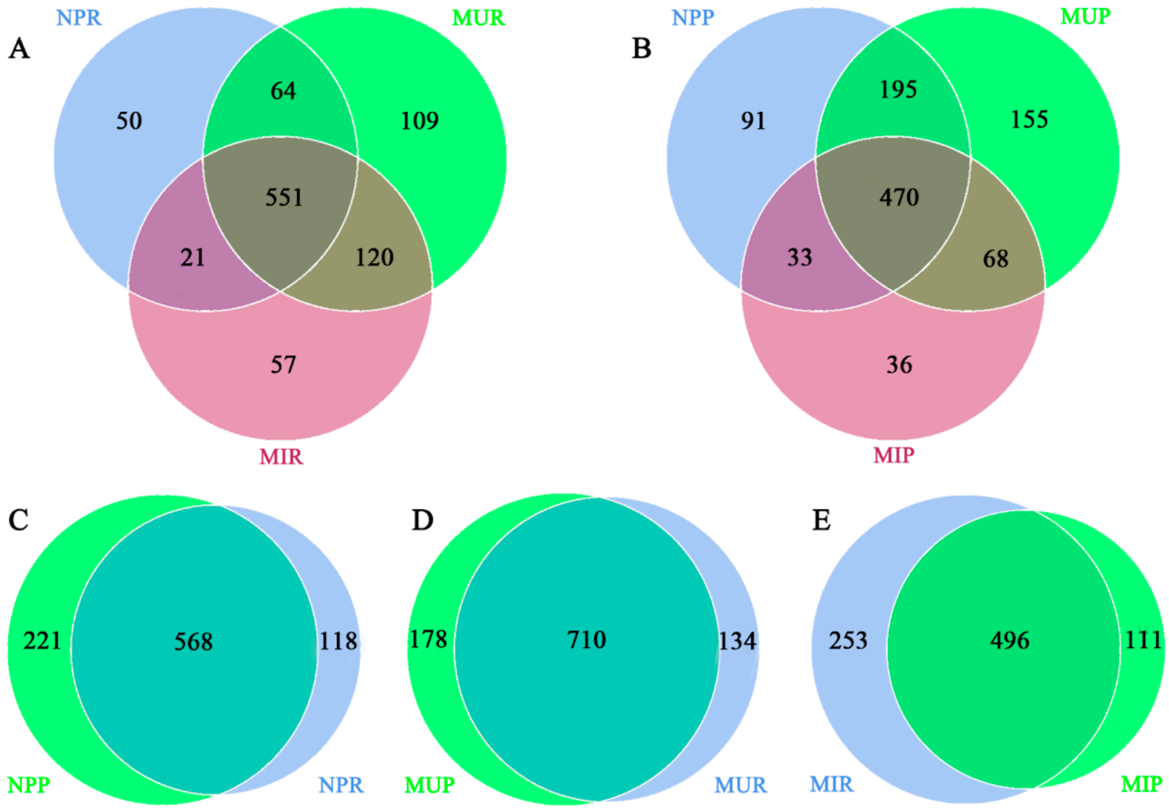

Figure S4. NPR, MUR and MIR represent the rhizosphere soil from the newly planted, monocultured uninfected and infected plants, respectively. NPP, MUP and MIP represent the rhizoplane soil from the newly planted, monocultured uninfected and infected plants, respectively.

International Journal of
Molecular Sciences

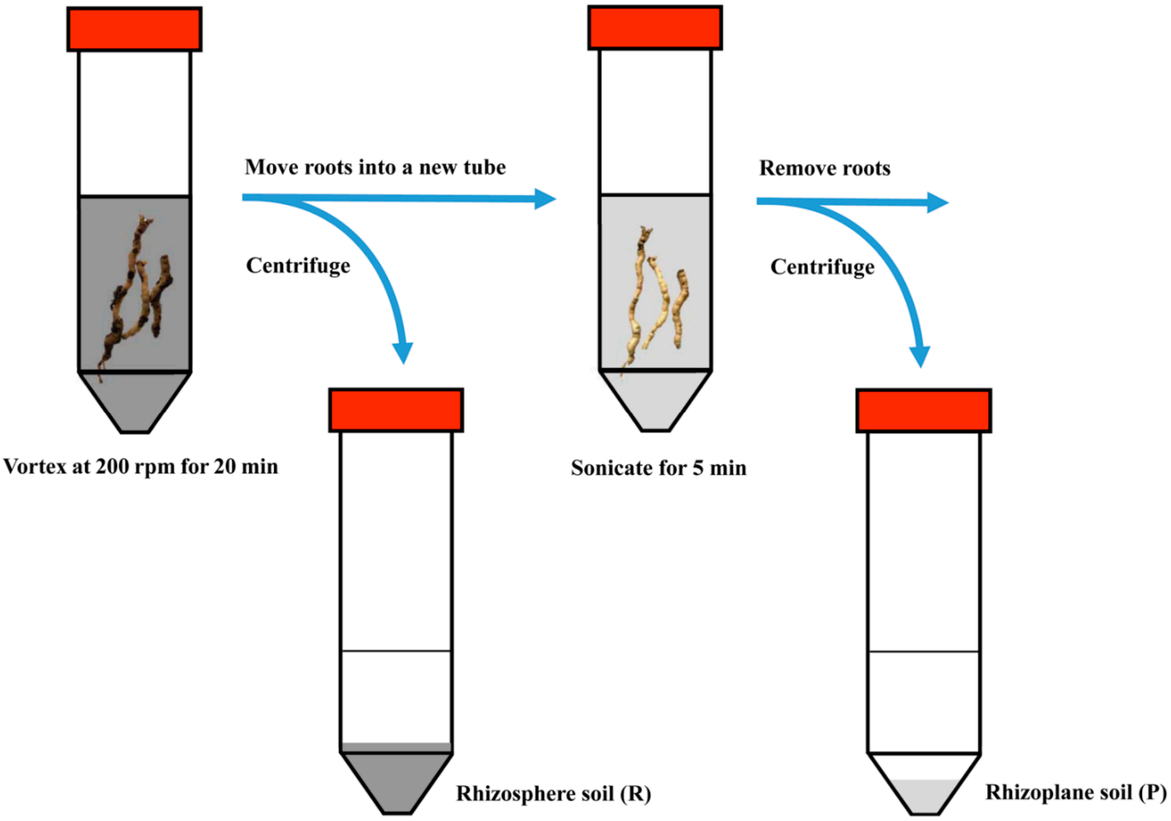

Figure S5. Scheme for the collection of rhizosphere and rhizoplane soils of *R. glutinosa*.

Table S1. The description of six soil categories.

Site code	Soil sample description
**Rhizosphere (R)**	
NPR	The rhizosphere soil from the newly planted (NP) group
MUR	The rhizosphere soil from the two-year monocultured uninfected (MU) group
MIR	The rhizosphere soil from the two-year monocultured infected (MI) group
**Rhizoplane (P)**	
NPP	The rhizoplane soil from the newly planted (NP) group
MUP	The rhizoplane soil from the two-year monocultured uninfected (MU) group
MIP	The rhizoplane soil from the two-year monocultured infected (MI) group

Table S2. The numbers of effective sequences that could be assigned to each taxonomic level in 18 soil samples.

Sample name	Kingdom	Phylum	Class	Order	Family	Genus	Species
**NPR1**	59,208	59,202	59,170	59,116	58,905	49,322	14,934
**NPR2**	61,110	61,107	61,042	60,986	60,740	50,552	15,311
**NPR3**	51,977	51,971	51,940	51,875	51,669	43,185	12,711
**MUR1**	60,914	60,876	60,808	60,653	59,735	55,973	11,207
**MUR2**	58,414	58,378	58,341	58,207	57,338	53,860	10,708
**MUR3**	56,888	56,848	56,797	56,654	55,732	52,218	10,267
**MIR1**	62,416	62,411	62,369	62,328	61,849	59,438	12,914
**MIR2**	59,747	59,742	59,714	59,658	59,154	56,792	12,392
**MIR3**	55,966	55,958	55,909	55,852	55,394	53,198	11,698
**NPP1**	50,914	50,913	50,878	50,800	50,455	45,006	12,444
**NPP2**	56,324	56,317	56,298	56,193	55,773	49,928	14,162
**NPP3**	51,029	51,026	50,976	50,873	50,507	44,864	12,778
**MUP1**	47,176	47,153	47,134	46,999	45,898	41,950	8,584
**MUP2**	46,420	46,403	46,384	46,224	45,064	41,277	8,218
**MUP3**	50,733	50,708	50,675	50,506	49,306	44,929	9,352
**MIP1**	44,256	44,256	44,226	12,866	12,582	11,475	2,409
**MIP2**	60,108	60,104	60,082	19,473	19,030	17,430	3,608
**MIP3**	62,347	62,344	62,324	16,653	16,279	14,497	3,017
***Percentage**	100.00%	99.98%	99.91%	87.95%	86.89%	78.91%	18.75%

NPR, MUR and MIR represent the rhizosphere soil from the newly planted, monocultured uninfected and infected plants, respectively. NPP, MUP and MIP represent the rhizoplane soil from the newly planted, monocultured uninfected and infected plants, respectively. * This represents the average percentage of effective sequences from 18 soil samples that could be assigned to each taxonomic level.

Table S3. Species richness and diversity indices in different soil categories.

Treatments	Observed species	Chao1	ACE	Shannon
**The newly planted plants**				
NPR	495.7b	566.4a	593.7a	5.042b
NPP	575.0a	634.3a	652.6a	5.735a
**The monocultured uninfected plants**				
MUR	654.7b	718.5a	742.5a	5.675b
MUP	696.3a	755.9a	766.7a	5.975a
**The monocultured infected plants**				
MIR	555.3a	628.3a	651.9a	5.493a
MIP	431.7b	615.6a	565.8a	2.496b

NPR, MUR and MIR represent the rhizosphere soil from the newly planted, monocultured uninfected and infected plants, respectively. NPP, MUP and MIP represent the rhizoplane soil from the newly planted, monocultured uninfected and infected plants, respectively. Different letters in columns show significant differences between rhizosphere and rhizoplane (NPR vs. NPP; MUR vs. MUP; MIR vs. MIP), determined by Tukey's test ($p < 0.05$).
